# Experiments with Bibron’s Antidote to the Poison of the Rattlesnake

**Published:** 1858-06

**Authors:** 


### ECLECTIC DEPARTMENT,

### AND SPIRIT OF THE MEDICAL PERIODICAL PRESS.

---

*Experiments with Bibron's Antidote to the Poison of the Rattlesnake.* By WILLIAM A. HAMMOND, M. D., Assist. Surg. U. S. Army.

Some four years since, Prince Paul, of Wurtemberg, the celebrated naturalist, communicated to my friend, Mr. De Vesey, the results of some experiments performed before the French Academy of Sciences by Professor Bibron, relative to an antidote to the poison of a rattlesnake. According to Prince Paul, Professor Bibron allowed a rattlesnake to bite him in the lips, cheeks, &c., and, by taking the antidote discovered by him, prevented all alarming symptoms, and, in fact, suffered no inconvenience therefrom.

The antidote in question, as stated by Prince Paul, is prepared according to the following recipe: R.—Potassii iodidi gr. iv; hydrarg. chl[illegible]ridi corros. gr. ij; bromini ℥v.—M. Ten drops of this mixture diluted with a tablespoonful or two of wine or brandy constitute a dose, to be repeated if necessary. It must be kept in glass-stoppered vials well secured.

Prince Paul forwarded a small quantity of the above mixture to Mr. De Vescy, who used it successfully in the cases of two men bitten by rattlesnakes near his residence in Iowa.

During a recent expedition to the Rocky Mountains, I had several opportunities of testing its efficacy, and, since my return, have performed additional experiments with it. The results have been, on the whole, exceedingly satisfactory, and I think that when taken in time it may be entirely depended upon in the poisonous wounds of the rattlesnake, and, perhaps, also in those of other venomous serpents.

1*st Experiment.* Heinrich Brandt, Acting Hospital Steward, was bitten, on the 2d of July, 1857, in the index finger of the right hand by a large rattlesnake (*crotalus confluentus,*) which he was in the act of putting into a jar for preservation. The snake inflicted a very deep wound, and hung by his fangs to the finger for a second or two before it could be detached. About four months after the bite, and before much pain or swelling had ensued, I administered one dose of Bibron's antidote. The symptoms almost immediately disappeared. Forty minutes after giving the first dose the pain and swelling returned, attended with considerable throbbing. I repeated the medicine, and in less than five minutes the finger had regained its natural appearance, and all pain and pulsation had vanished. He remained perfectly well, and resumed his duties in an hour from the reception of the injury.

2*d Experiment.* A very large rattlesnake was made to bite a young wolf (*Canis occidentalis,*) about three months old. The serpent wounded the animal severely in the left flank. Fifteen minutes after the bite the leg was much swollen, and the wolf exhibited signs of great uneasiness, yawning, stretching, and looking about in an anxious manner. These symptoms continued to increase in intensity till inability to stand, drowsiness, and slight convulsive movements ensued. I now (thirty minutes from the infliction of the wound,) gave six drops of the antidote, with almost instantaneous disappearance of the observed symptoms. In a few minutes afterwards the animal ate a large piece of meat.

3*d Experiment.* On the following day the same snake was made to bite the wolf three times in the space of five minutes, in the flank, neck and chest. In two minutes after the last bite the effects of the poison were evidenced by the inability of the wolf to stand, gasping respiration, and a fixed expression of counrenance. Some delay occurred in getting the antidote ready, and before I could administer it all signs of life had apparently ceased. Nevertheless, I placed six drops far down the throat, where it seemingly remained, as no effort of swallowing was perceived. However, in one minute respiration again commenced, and the heart could be felt to pulsate. The wolf lived for twenty-seven minutes, and then died comatose.

The rapidity of the action of the poison in this case, owing to the large quantity introduced into the system, prevented a successful issue. The good effects of the antidote were, however, sufficiently apparent to every observer, and I have no doubt that had it been given before the faculty of swallowing was lost, the life of the animal would have been saved.

4*th Experiment.* After my return to Fort Riley, a large *Crotalus confluentus*, which I had brought with me from the Rocky Mountains, was made to bite a dog five months old. The wound was made in the right shoulder. The poisonous effects of the bite commenced in ten minutest causing gasping respiration, inability to stand, &c. I attempted to give a dose of the antidote, but the dog would not swallow, and I had no means at hand by which to introduce it into the stomach. I again tried to administer the remedy, but without success. The third dose was inhaled into the lungs. By this time the dog was perfectly senseless, and was dead in forty-five minutes after the infliction of the bite. Very slight swelling occurred in the wounded part.

5*th Experiment.* Forty-five minutes after the last experiment the same snake was made to bite another dog of the same litter as the preceding. The wound was inflicted in the lower jaw, very near the mouth. At the end of three minutes, and before any violent symptoms ensued, a dose of the antidote was given. The dog swallowed it readily. Five minutes afterwards the animal seemed very uneasy. Respiration was accelerated, and he preferred to lie down in the shade. At the end of about fifteen minutes he could stand with difficulty; and, as the sickness appeared to be on the ncrease, another dose was administered. Nearly half of this was lost.

Slight swelling was perceived in the face and neck. When roused the animal would walk a few yards, though with great difficulty, and evidently preferred rest and quiet. About an hour after the bite he lapped a little milk, and seemed to be better, wagging his tail when spoken to, and walking with less effort. No increase of the symptoms occurred, and, in fact, the dog was, to all appearance, perfectly well in two hours after the reception of the injury, except that a slight swelling of the under jaw still remained. I saw him no more till next morning, when this had disappeared, and he was as active and lively as ever.

I had no further opportunities of repeating the experiments with other animals. During my absence, however, the antidote was use by Dr. Coolidge, U. S. Army, (to whom I am also indebted for assistance in the latter experments,) in the following case, of which he has favored me with the subjoined account:

"In July, 1857, a girl, aged fifteen years, was bitten at Fort Riley by a rattlesnake, on the dorsal aspect of the first phalanx of the ring finger of the right hand. In a few moments the finger became swollen and bluish, and when I first saw her, about ten minutes after the receipt of the wound, I applied a cord tightly around the finger, and then made a free incision down to the bone. As soon as the articles could be procured from the hospital, I gave ten drops of the bromine mixture diluted, and injected into the wounded finger the preparation recommended by Dr. David Brainard, of Chicago, Illinois. (See *Annual Report*, *Smithsonian Institution*, 1854.) viz:

℞ Iodinii, grs. x.

Potasii iodidi, grs. xxx.

Aquæ destillatæ, f℥j. Solve.

The patient expressed herself relieved after the first dose of the bro mine; a second was given in twenty minutes. The solution of the iodine injected caused severe smarting pain; the fluid and air from the syringe could be felt a little above the wrist, and ultimately caused suppuration of the cellular tissue on the back of the hand. Nothing more was done. The girl recovered."

In conjunction with the mixture referred to in this paper, it will be observed that Dr. Coolidge laid open the wound and injected the cellular tissue with tinbture of iodine, as recommended by Dr. Brainard, of Chicago, so that the favorable result in this instance cannot be attributed solely to the use of Bibron's antidote.—*American Journal of Med. Sciences.*

